# Comparison of two distinct arrival and treatment programs for bovine respiratory disease in high-risk feeder cattle entering a feedlot

**DOI:** 10.1093/tas/txac102

**Published:** 2022-07-27

**Authors:** John P Pollreisz, Charley Cull, Kelly Lechtenberg, Thomas Short, Mitchell Blanding, Jess Hinrichs, Heather D Hughes

**Affiliations:** Zoetis, Parsippany, NJ 07054, USA; Midwest Veterinary Services, Inc., Oakland, NE 68045, USA; Midwest Veterinary Services, Inc., Oakland, NE 68045, USA; Zoetis, Parsippany, NJ 07054, USA; Zoetis, Parsippany, NJ 07054, USA; Zoetis, Parsippany, NJ 07054, USA; Zoetis, Parsippany, NJ 07054, USA; SciWrite Consulting LLC, Canyon, TX 79015, USA

**Keywords:** antimicrobial, bovine respiratory disease, cattle, metaphylaxis, mixed treatment comparison, net returns

## Abstract

Antimicrobial metaphylaxis of high-risk cattle entering the feedlot is a common management strategy implemented against bovine respiratory disease (**BRD**). Typically, following a prescribed postmetaphylactic interval (**PMI**), animals displaying clinical signs of BRD are pulled from the feedlot pen and treated with antimicrobials when treatment criteria are met. The objective of this study was to compare 2 distinct sequential BRD treatment protocols each consisting of a metaphylactic antimicrobial plus 2 potential subsequent as-needed treatment antimicrobials. Heifers at high-risk for BRD (*n* = 1000; initial BW = 229 kg ± 1.6) purchased from sale barns in the southeastern U.S. were transported to a contract research feedlot in Nebraska and randomly assigned to 1 of 2 experimental groups (10 blocks of 100 animals each; 50 per treatment group). Experimental groups consisted of: (1) tulathromycin metaphylaxis (2.5 mg/kg) followed by ceftiofur crystalline free acid (6.6 mg/kg) and danofloxacin (8 mg/kg) for subsequent first and second as-needed BRD treatment, respectively (**TCD)** or (2) tildipirosin metaphylaxis (4 mg/kg) followed by florfenicol-flunixin meglumine (40 mg/kg florfenicol; 2.2 mg/kg flunixin meglumine) and enrofloxacin (12.5 mg/kg) for subsequent first and second as-needed BRD treatment, respectively (**TFFE**). Following expiration of the 7-d PMI, calves that showed signs of clinical BRD were pulled and examined to determine if treatment was necessary based on a clinical attitude score (CAS). Heifers with a CAS of 1 accompanied by ≥40°C rectal temperature, and all heifers with a CAS ≥ 2 regardless of rectal temperature, received the appropriate first-treatment antimicrobial. Upon relapse, following expiration of the post-treatment interval (**PTI**), heifers received the appropriate second-treatment antimicrobial. In the first 90 d, calves in the TFFE experimental group received more first-treatments than calves in the TCD experimental group (*P* = 0.054) and resulted in 50% greater mortality (*P* < 0.043) relative to the TCD heifers. From d 0 to closeout, first-treatment morbidity as well as mortality were greater in TFFE relative to TCD (*P* ≤ 0.032). Growth performance did not differ between treatments in the first 90 d; however, ADG was greater (*P* = 0.033) and G:F improved (*P* = 0.014) at closeout in TCD versus TFFE on a deads-in basis. Closeout economics revealed a $50.78/animal greater profit in the TCD experimental group relative to TFFE.

## INTRODUCTION

Metaphylaxis is the administration of Food and Drug Administration (FDA)-approved antimicrobials to groups of animals at high-risk for bovine respiratory disease (BRD) to minimize acute onset of disease (Dennis et al., 2018). Various strategies are used to mitigate BRD in newly received high-risk cattle in feedlots, including administering metaphylaxis upon arrival, treating only animals with clinical symptoms upon arrival, or pulling and treating animals with clinical symptoms on a daily basis after arrival (United States Department of Agriculture, 2019). The term “high-risk” cattle generally refers to cattle with one or more risk factors for BRD, such as unknown health history, unknown source, transportation stress, or recent weaning (Nickell and White, 2010; Ives and Richeson, 2015; Smith et al., 2017), and metaphylaxis is implemented by 39% of U.S. feedlots (*n* ≥ 1000) on 17% of cattle (United States Department of Agriculture, 2019).

Several antimicrobials are approved for metaphylaxis for BRD, and the selection for use is based upon efficacy and cost-effectiveness of these products (Nickell and White, 2010). Numerous clinical trials have been conducted to assess the efficacy of various antimicrobials for the treatment and control of BRD in high-risk cattle (DeDonder and Apley, 2015; Ives and Richeson, 2015).

Macrolides are a class of antibiotics often used for metaphylaxis in feedlots and are long-acting injectable solutions which have label claims for the control and treatment of BRD (Kinnear et al., 2020). The mode of action of these compounds involves disrupting bacterial protein synthesis (Zaheer et al., 2013). Two such antimicrobials are tulathromycin and tildipirosin, which have each demonstrated effectiveness at mitigating morbidity and mortality in high-risk feedlot cattle (Abell et al., 2017); however, there are numerous approaches to controlling and treating BRD related to the metaphylactic antimicrobial chosen by the producer and their veterinarian, subsequent antimicrobials/anti-inflammatories chosen for any necessary retreatments, and their combinations.

Following metaphylaxis, a post-metaphylactic interval (PMI; typically 5-10 d) is implemented prior to administering any necessary retreatments of animals displaying clinical BRD symptoms of fever and/or depression (United States Department of Agriculture, 2019). In populations of cattle in North American feedlots that have received antimicrobial metaphylaxis, approximately 15% of feedlot cattle require a second antimicrobial treatment for disease (Sanderson et al., 2008; APHIS. USDA, 2013; Miles and Rogers, 2014). Within those retreated cattle, 90% were reported to have received retreatment with antimicrobials of a different mechanistic class (APHIS. USDA, 2013). However, studies examining the comparative effects of various complete BRD treatment protocols (metaphylactic antimicrobial plus specific retreatment antimicrobials) on health, growth, and economic outcomes are scarce (Coetzee et al., 2019).

Therefore, the objectives of the current study were to compare 2 sequential BRD antibiotic management protocols; the first (TCD), comprised of tulathromycin metaphylaxis (Draxxin, Zoetis, Parsippany, NJ) followed by ceftiofur crystalline free acid (Excede, Zoetis, Parsippany, NJ) followed by danofloxacin (Advocin, Zoetis, Parsippany, NJ) for subsequent as-needed individual treatments, compared to a corresponding protocol (TFFE) comprised of tildipirosin metaphylaxis (Zuprevo, Merck Animal Health, Madison, NJ) followed by florfenicol-flunixin meglumine (Resflor Gold, Merck Animal Health, Madison, NJ) followed by enrofloxacin (Baytril, Elanco Animal Health, Greenfield, IN) for subsequent as-needed individual treatments for BRD. The intent was to evaluate the 2 BRD antibiotic management protocols for their ability to limit the development and expression of BRD in high-risk feeder cattle entering the feedlot, measured as total treatments for BRD and mortality, as well as the impact these strategies have on growth performance and economics through the end of the feeding period.

## MATERIALS AND METHODS

This study was conducted from November 2019 to August 2020 in a research feedlot facility in Nebraska and followed an approved protocol whereby routine management practices of the commercial feedlot are in accordance with 7 U.S. Code 54.

### Cattle Arrival

Heifers at high risk of BRD (*n* = 1,000; initial BW = 229 kg ± 1.6), acquired from sale barns in Tennessee, were transported approximately 20 h to a contract research feedlot facility in Nebraska. Animals were received between November 4, 2019 and November 21, 2019 and were randomized to 1 of 2 treatments utilizing a pre-determined randomization schedule as cattle entered the squeeze chute. Truck-load lots of heifers were acquired until an adequate number of cattle were purchased to fill the specified number of pens to the correct animal density (50 animals/pen; 2 pens/block). All heifers from each truck load had an equal chance of being allocated to either of the 2 experimental groups and were processed 24 h after arrival, placed in holding pens with access to meadow hay and ad libitum access to water, and assigned to a home pen from a set of 2 adjacent home pens. Postprocessing, each block of 2 treatments was moved to its designated set of pens.

Each animal was assigned a unique number in the form of duplicate numbered ear tags, 1 for each ear. If a tag was lost, it was replaced immediately. Tag color was identical for all cattle within a pen and differed from cattle in adjoining pens to facilitate rapid identification of cattle in incorrect pens after study initiation.

All cattle used in the study were owned by the research facility. The clinical investigator for the research site was responsible for the welfare of the animals during the study. Heifers with a clinical score of 3 or 4 prior to d 0 (Table 1) and/or heifers exhibiting any concurrent disease or physical conditions that might interfere with the progression of BRD were subject to exclusion from the study.

**Table 1.** Clinical attitude score system

CAS^1^	Classification	Brief description
0	None	No BRD^2^ clinical signs
1	Mild	Mild depression; slower in movement but no signs of weakness; small amount of serous nasal discharge
2	Moderate	Moderate depression; signs of weakness or “knuckling” and calf may be reluctant to stand or move about pen; some shallowness apparent in left flank; considerable serous nasal discharge or moderate amount of mucopurulent nasal discharge; dyspnea or respiratory rate is increased; cough or coughing episodes are present.
3	Severe	Severe depression; stumbling or moves with extreme prodding; obvious lack of fill in left flank signaling anorexia; head lowered or extended to facilitate breathing; may be open-mouthed breathing with considerable noise (expiratory grunts, moans); copious mucopurulent to purulent nasal discharge; cough or coughing episodes are present.
4	Moribund	Calf is moribund and near death; calf in general is not ambulatory, cannot rise from recumbency, and cannot acquire food or water. Very likely cannot be removed from pen for treatment without mechanical transport. Animal must be euthanized.

Clinical attitude score.

Bovine respiratory disease.

Animals were housed in typical U.S. commercial feedlot pens (15.2 m wide by 76.2 m long; 0.305 m bunk space per animal) with dirt floors and mounds. The photoperiod was typical of the normal seasonal duration in the study period from November 2019 to August 2020.

### Experimental Design and Treatments

The study was conducted using a randomized complete block experimental design in which blocks of cattle were defined based on source, date of arrival to the feedlot, and study start date. Pen served as the experimental unit to evaluate both health and growth performance variables. Pairs of pens were considered a block, each consisting of 100 animals, 50 per treatment group. Each block consisted of a truck-load of cattle, received and processed on the same day, and blocks of cattle completed the study on the same day. Cattle were purchased in 100 animal lots for this experiment. End point of the study was at cattle harvest.

Treatments consisted of: (1) tulathromycin metaphylaxis (Draxxin, Zoetis, 2.5 mg/kg BW) followed by ceftiofur crystalline free acid (Excede, Zoetis, 6.6 mg/kg BW) and danofloxacin (Advocin, Zoetis, 8 mg/kg BW) for subsequent first and second as-needed BRD treatment, respectively (TCD) or (2) tildipirosin metaphylaxis (Zuprevo, Merck Animal Health, 4 mg/kg BW) followed by florfenicol-flunixin meglumine (Resflor Gold, Merck Animal Heatlh, 40 mg/kg BW florfenicol; 2.2 mg/kg BW flunixin meglumine) and enrofloxacin (Baytril, Elanco Animal Health, 12.5 mg/kg BW) for subsequent first and second as-needed BRD treatment, respectively (TFFE). Treatment assignment is illustrated in Table 2.

**Table 2.** Treatment assignment

Treatment	Product	Metaphylaxis dosage	Post-metpahylactic interval, d	Therapeutic dosage	Post-treatment interval, d	Pens
TCD^1^	Tulathromycin^3^	2.5 mg/kg	7	N/A	N/A	10
	Ceftiofur crystalline free acid^4^	N/A	N/A	6.6 mg/kg	5	
	Danofloxacin^5^	N/A	N/A	8 mg/kg	3	
TFFE^2^	Tildipirosin^6^	4 mg/kg	7	N/A	N/A	10
	Florfenicol-flunixin meglumine^7^	N/A	N/A	40 mg/kg florfenicol; 2.2 mg/kg flunixin meglumine	5	
	Enrofloxacin^8^	N/A	N/A	12.5 mg/kg	3	

Metaphylaxis with tulathromycin (2.5 mg/kg BW), followed by first BRD treatment with ceftiofur crystalline acid (6.6 mg/kg BW), followed by second BRD treatment with danofloxacin (8 mg/kg BW).

Metaphylaxis with tildipirosin (4 mg/kg BW), followed by first BRD treatment with florfenicol-flunixin meglumine (40 mg/kg BW; 2.2 mg/kg BW), followed by second BRD treatment with enrofloxacin (12.5 mg/kg BW).

Draxxin, Zoetis, Parsippany, NJ.

Excede, Zoetis, Parsippany, NJ.

Advocin, Zoetis, Parsippany, NJ.

Zuprevo, Merck Animal Health, Madison, NJ.

Resflor Gold, Merck Animal Health, Madison, NJ.

Baytril, Elanco Animal Health, Greenfield, IN.

### Cattle Processing

At arrival processing (considered d 0) and throughout the study, animals were evaluated using a Clinical Attitude Scoring (CAS) system (Table 1), regardless of treatment assignment. Personnel with appropriate training and experience processed all cattle during the study. An ear notch was taken at initial processing and submitted for determination of persistent infection with bovine viral diarrhea virus (BVD-PI) and positives removed from the study upon identification (Table 3).

**Table 3.** Observations associated with health-related variables

Item, hd^1^	TCD^2^	TFFE^3^
Total retreated	302	318
First 90 d		
Total individual treatments	191	237
First BRD^4^ treatment	145	177
Second BRD treatment	46	60
BRD deaths	39	61
Euthanized for BRD	3	4
d 91 to closeout		
Total individual treatments	5	12
First BRD treatment	3	8
Second BRD treatment	2	4
BRD deaths	4	9
Euthanized for BRD	0	1
Total BRD deaths (d 0 to closeout)	43	70
Final disposition		
BVD-PI^5^	1	1
Lame	1	–
Euthanized other than BRD	–	1
Death BRD	40	65
Euthanized for BRD	3	5
Slaughter	455	428
Total	500	500

Head.

Metaphylaxis with tulathromycin (2.5 mg/kg BW), followed by first BRD treatment with ceftiofur crystalline acid (6.6 mg/kg BW), followed by second BRD treatment with danofloxacin (8 mg/kg BW).

Metaphylaxis with tildipirosin (4 mg/kg BW), followed by first BRD treatment with florfenicol-flunixin meglumine (40 mg/kg BW; 2.2 mg/kg BW), followed by second BRD treatment with enrofloxacin (12.5 mg/kg BW).

Bovine respiratory disease.

Persistent infection with bovine viral diarrhea virus.

Animals assigned to TCD treatment were weighed and treated with tulathromycin based on observed body weight (2.5 mg/kg BW), and treatment information was recorded. Injections of the anti-infectives used in this study were administered subcutaneously (SC) in the lateral neck with a disposable syringe graduated to at least 0.2 mL increments and disposable needles. Likewise, animals assigned to TFFE experimental group were weighed and treated with tildipirosin according to observed body weight (4 mg/kg BW), SC in the lateral neck with dosage recorded.

Other standard feedlot arrival preventative measures were applied to protect against bovine respiratory disease (Bovi-Shield GOLD 5, administered SC in the neck region, Zoetis, Parsippany, NJ; 2 mL/animal), clostridial diseases (Ultrachoice 7, 2 mL administered SC in the neck region, Zoetis, Parsippany, NJ), and parasites (Dectomax injectable, 200 mcg/kg BW administered SC in the neck region and Valbazen suspension, 10 mg/kg BW administered orally, Zoetis, Parsippany, NJ). Cattle also received a growth implant containing 200 mg testosterone proprionate and 20 mg estradiol benzoate administered SC in the middle one-third of the back of 1 ear at initial processing (Synovex H; Zoetis, Parsippany, NJ). As animals exited the squeeze chute, they were placed in designated pens according to treatment assignment. Cattle received a second implant of 200 mg trenbolone acetate and 28 mg estradiol benzoate administered SC in the middle one-third of the ear (Synovex One, Zoetis, Parsippany, NJ) on d 90.

### Feed Management

Heifers were offered standard receiving, step-up, and feedlot finishing diets as appropriate, which were formulated to meet or exceed National Academies of Sciences, Engineering, and Medicine (NASEM) Nutrient Requirements of Beef Cattle (2016) according to class and weight. Water was available ad libitum. No changes in feed management were made between blocks on the study.

Heifers were offered receiving diets at the time of arrival to the feedlot, formulated to meet or exceed NASEM (2016) requirements. Diet ingredients, including generic or trade labels were included in the study documentation. Diet changes were per facility procedures and documented. The diet included an ionophore, monensin (Rumensin 90, Elanco Animal Health, Greenfield, IN; ranging between 100 mg·heifer^−1^·d^−1^ at study initiation and 415 mg·heifer^−1^·d^−1^ at study endpoint), for the prevention and control of coccidiosis and improved feed efficiency; however, the diet did not contain antimicrobials that would affect BRD therapy.

Diets formulated to meet or exceed NASEM (2016) requirements for class and weight were used to gradually advance heifers to final feedlot finishing diets. Diet ingredients and diet changes were documented in the study files. Monensin (Rumensin, Elanco Animal Health) was administered for the duration of the study, tylosin phosphate (Tylan 100, Elanco Animal Health, Greenfield, IN; 81 mg·heifer^−1^·d^−1^) was administered for approximately the last 7 mo of the study for liver abscess control, and melengesterol acetate (MGA, Zoetis, Parsippany, NJ; 0.5 mg·heifer^−1^·d^−1^) was administered for estrus control for the last 120 d on feed. Step-up diets were approximately 62% dry matter, and the final diet (last 120 d) contained 68% dry matter.

Diets were mixed daily. Scales and/or feed mixers used to weigh feed ingredients were certified or approved within approximately 180 d of study initiation. Feed mixing details for each batch and amount delivered at each feeding were documented in the study file. Feed left in the bunk (orts) was removed if off condition and quantified at the end of the study period.

### Animal Weights

Cattle were individually weighed on d 0 for the purpose of ascertaining dosage weights for their respective treatments and documented. Scale checks for accuracy were performed according to site standard operating procedures and at a minimum, on each d that BW was obtained.

At the end of the first phase (~d 90), individual BW was ascertained to measure gain during this period. Final pen weights rather than individual BW were taken at the completion of the study at the time of shipment for harvest. Additionally, any animal that was pulled for treatment (first or second BRD treatment) was individually weighed to establish treatment dosage. Animals removed from the study or that died during the study were weighed upon removal and recorded for documentation.

### Animal Health Management

Animals were observed individually each day by personnel masked to treatments and abnormal observations were recorded. The CAS index (Table 1) was used for BRD evaluation and documentation of CAS score and BRD-related observations retained. Non-BRD related observations were recorded and considered as possible adverse events and recorded if appropriate.

Following expiration of the 7-d PMI, calves that showed clinical signs of BRD were pulled and examined to determine if treatment was necessary. Calves with a CAS of 1 accompanied by ≥40°C rectal temperature were treated with the appropriate BRD first-treatment antimicrobial according to study treatment group (TCD received ceftiofur crystalline free acid; TILD received florfenicol-flunixin meglumine). Calves with clinical signs consistent with a CAS of 2, regardless of body temperature, received the appropriate BRD first-treatment antimicrobial according to experimental group. Cattle that relapsed following expiration of the BRD first-treatment post-treatment interval (PTI) were treated by the same CAS and rectal temperature criteria with the appropriate BRD second-treatment antimicrobial according to experimental group (TCD received danofloxacin; TFFE received enrofloxacin). Calves were returned to home pens immediately following retreatment. Animals that received two antibiotic treatments for BRD following metaphylaxis were considered chronically ill but were not removed from the home pen. These animals were not pulled for further individual treatment for BRD.

At any point during the study, animals requiring emergency intervention with a CAS of 3 were treated regardless of timing relative to PMI or PTI, according to the research facility’s standard operating procedure, and remained on the study. A necropsy was performed on all BRD mortalities, and a representative lung sample was hand-delivered to the on-site laboratory, Central States Research Centre, Inc. for culture. Any animal with a CAS of 4 was humanely euthanized and a necropsy was performed to assess pathology and determination of cause of death. Animals that were euthanized with confirmation of BRD were included in the results as part of BRD mortality. In all cases, the investigator or study veterinarian was responsible for assuring that animal welfare concerns were addressed. Animals were removed from the study if moribund or if the clinical investigator’s judgment was such that severity and/or duration of the health condition of the animal would affect normal growth (e.g., a broken leg). Animals removed from the study were not returned to the experimental pen and detailed explanation for removal was documented. No abnormal health observation was considered a “Suspected Adverse Product Experience” in the current study.

### Statistics

Morbidity, mortality, and growth performance data (ADG and G:F) were analyzed with pen as experimental unit in a randomized complete block design using SAS (9.4, SAS Institute, Cary, NC). All growth performance variables were analyzed as both ‘deads and removals included’ (deads-in) and ‘deads and removals excluded’ (deads-out; Table 5). For analysis of deads-in growth performance data, animals were included up to the point that they were removed from the experiment, and for deads-out growth performance data, deads and removals were completely excluded from the analysis. Primary and secondary response criteria included treatments for BRD following metaphylaxis (first BRD treatment, treated = 1, untreated = 0), second BRD treatment (treated = 1, untreated = 0) and BRD mortality (death = 1, survive = 0) and were analyzed using a generalized linear mixed model with a binomial error distribution and a logit link function. The model included the fixed effect of BRD treatment and random effect of block. The interaction of BRD treatment by block was included in the model to represent pen effect. Back-transformed least squares means and 95% confidence intervals were constructed by treatment and treatment differences assessed at the 5% (2-sided) level of significance.

**Table 5.** Growth performance and feed efficiency (G:F)

Item	TCD^1^	TFFE^2^	SE	*P*-value
d 0 to 90				
Initial weight, kg	228.9	228.5	1.60	0.776
d 90 weight, kg	347.8	347.9	3.40	0.988
Deads-out ADG^3^, kg/d	1.29	1.28	0.04	0.932
Deads-in ADG, kg/d	1.06	0.90	0.10	0.102
G:F^4^, deads out	0.17	0.17	0.06	0.712
G:F, deads in	0.13	0.09	0.85	0.114
d 91 to closeout				
Deads-out ADG, kg/d	1.23	1.21	0.03	0.580
Deads-in ADG, kg/d	1.21	1.18	0.02	0.262
G:F, deads-out	0.14	0.14	0.10	0.282
G:F, deads-in	0.14	0.14	0.07	0.114
d 0 to closeout				
Deads-out ADG, kg/d	1.24	1.21	0.02	0.313
Deads-in ADG, kg/d	1.16	1.08	0.03	0.033
G:F, deads-out	0.15	0.15	0.04	0.193
G:F, deads-in	0.14	0.13	0.10	0.014
Shipping weight, kg	574.0	573.1	3.80	0.842

Metaphylaxis with tulathromycin (2.5 mg/kg BW), followed by first BRD treatment with ceftiofur crystalline acid (6.6 mg/kg BW), followed by second BRD treatment with danofloxacin (8 mg/kg BW).

Metaphylaxis with tildipirosin (4 mg/kg BW), followed by first BRD treatment with florfenicol-flunixin meglumine (40 mg/kg BW; 2.2 mg/kg BW), followed by second BRD treatment with enrofloxacin (12.5 mg/kg BW).

Average daily gain.

Gain:feed (DM basis).

## RESULTS AND DISCUSSION

Table 3 shows number of cattle pulled, treated, and dead, as well as final disposition of all calves enrolled in the study. Two calves (1 in each treatment group) were BVD-PI positive based on testing at enrollment and removed from the study on d 9. One animal in the TCD group was removed at d 63 due to lameness and 1 animal in the TFFE group was euthanized and removed at d 205 due to a broken leg but was treated once for BRD and included as a first BRD treatment in final health outcomes. Four hundred fifty-five and 428 heifers were present at harvest at the completion of the study in the TCD and TFFE treatment groups, respectively.

In the first 90 d of the study, when cattle are most susceptible to BRD (Taylor et al., 2010), the TCD group required less first treatments for BRD than the TFFE group (*P* = 0.054; Table 4). Metaphylaxis is effective in reducing feedlot morbidity and mortality (Dennis et al., 2020), but efficacy is dependent upon antimicrobial used, cattle placement weight, health risk, location, and season. Results of this study indicate that tulathromycin was more effective at controlling disease in newly received feedlot heifers at high risk for BRD than tildipirosin, particularly in the first 90 d when cattle are most susceptible to infection with BRD (Taylor et al., 2010). Multiple studies have examined the comparative efficacy of tulathromycin versus tildipirosin used for metaphylaxis. In newly weaned crossbred steers considered high-risk (initial BW = 285.4 kg), tulathromycin for metaphylaxis on arrival lowered BRD morbidity, decreased the number of chronic animals, and reduced overall mortality at closeout relative to tildipirosin (Sturgess and Renter, 2017). Furthermore,O’Conner et al. (2013) reported reduced risk for retreatment of cattle administered tulathromycin in a mixed treatment comparison meta-analysis of antimicrobial treatments for undifferentiated BRD when compared to tildipirosin.

**Table 4.** Morbidity and mortality

Item	TCD^1^	TFFE^2^	SE	*P*-value
d 0 to 90				
FirstBRDtreatments, %	27.8	34.4	4.51	0.054
Metaphylaxis success rate, %	72.2	65.6	4.51	0.054
SecondBRDtreatments, %	8.5	11.1	2.12	0.182
Second BRD treatments as a % of first	31.5	32.9	4.65	0.802
BRD^3^ Mortality, %	6.5	10.4	2.32	0.043
d 91 to closeout				
FirstBRDtreatments, %	0.6	1.6	0.45	0.179
SecondBRDtreatments, %	0.4	0.8	2.34	0.443
BRD mortality, %	0.8	1.7	4.86	0.217
d 0 to closeout				
FirstBRDtreatments, %	28.5	36.1	4.44	0.032
Temperature at first treatment, C°	40.2	40.2	0.17	0.671
Clinical attitude score^4^	1.40	1.41	0.063	0.984
Second BRD treatments, %	8.8	11.8	2.25	0.141
Total BRD treatments	39.2	49.8	3.00	0.082
BRD mortality, %	7.4	12.4	2.40	0.023
BRD case fatality rate, %	27.9	43.0	7.34	0.120

Metaphylaxis with tulathromycin (2.5 mg/kg BW), followed by first BRD treatment with ceftiofur crystalline acid (6.6 mg/kg BW), followed by second BRD treatment with danofloxacin (8 mg/kg BW).

Metaphylaxis with tildipirosin (4 mg/kg BW), followed by first BRD treatment with florfenicol-flunixin meglumine (40 mg/kg BW; 2.2 mg/kg BW), followed by second BRD treatment with enrofloxacin (12.5 mg/kg BW).

Bovine respiratory disease.

A score ranging from 0 (normal) to 4 (moribund) based on clinical and behavioral signs of BRD.

More recently, Abell et al. (2017) conducted a multiple treatment comparison meta-analysis (37 trials; 8 different antimicrobials) in which various metaphylactic antimicrobials were evaluated to quantify odds ratios of morbidity and mortality. The authors used results to designate antimicrobials as either “upper tier”, “middle tier” or “lower tier” based upon the odds of morbidity and mortality outcomes, and determined that tulathromycin performed as un “upper tier” antimicrobial, producing the lowest odds of BRD morbidity and mortality relative to all other metaphylactic antimicrobials, while tildipirosin performed as a “middle tier” antimicrobial. The superiority of tulathromycin was also evident in studies which compare it to other antimicrobials (gamithromycin and/or tilmicosin) for the control of BRD (Tennant et al., 2014; Miller et al., 2016). While the aforementioned studies demonstrate the efficacy of tulathromycin relative to tildipirosin, it is important to note that two distinct antibiotic BRD management protocols consisting of three antimicrobials each are being compared in the current study, rendering it presumptive to conclude that the effectiveness of each initial metaphylactic on its own is responsible for observed differences between experimental treatment groups, as opposed to the efficacy of each protocol in its entirety.

Forty-six heifers (8.5%) required a second BRD treatment in the TCD group relative to 60 (11.1%) in the TFFE group (*P* = 0.182). The second BRD treatment percentage of the 2 groups did not differ (*P* = 0.802). The comparative efficacy of ceftiofur crystalline free acid and florfenicol-flunixin meglumine is not well-established. Hannon et al. (2009) conducted a field trial in beef calves that received metaphylaxis with oxytetracycline upon arrival and were treated with either ceftiofur crystalline free acid or florfenicol-flunixin meglumine. Florfenicol-flunixin meglumine treated cattle exhibited lower mortality rates than ceftiofur crystalline free acid treated cattle. Another study compared the efficacy of ceftiofur crystalline free acid and florfenicol-flunixin meglumine for undifferentiated fever treatment following metaphylaxis with tulathromycin and resulted in no differences in overall chronic animals, salvage slaughter, or mortality rates between the two groups (Behlke et al., 2015).

While BRD morbidity and mortality typically occur in the first 60 to 90 d on feed in a feedlot, there were an additional 11 animals that were first treated between d 91 and closeout with 3 in the TCD group and 8 in the TFFE group (*P* = 0.179). There were 2 second BRD treatments in the TCD group and 4 second BRD treatments in the TFFE group (*P* = 0.433). Four animals in the TCD group and 9 in the TFFE group died in this second phase of the study (*P* = 0.217).

In the TFFE group, there was 50% greater mortality (*P* = 0.043) than in the TCD group in the first 90 d of the study. Collectively, from d 0 to closeout, first treatment morbidity was greater (*P* = 0.032) in the TFFE heifers compared to TCD heifers. Second BRD treatments over the course of the study were not different between treatment groups (*P* = 0.141); however, BRD mortality was significantly greater (*P* = 0.023) in the TFFE group versus the TCD group. Combined first and second BRD treatments, excluding metaphylaxis, from d 0 to closeout tended to be greater (*P* = 0.082) in TFFE cattle relative to TCD cattle. Bovine respiratory disease case fatality rate was numerically lower for the TCD group vs. the TFFE group (*P* = 0.120; 27.9% vs. 43%, respectively).

Growth performance is demonstrated in Table 5. Average initial BW (*P* = 0.776) and weights at the end of the first 90 d (*P* = 0.988) did not differ between treatment groups. Likewise, resulting ADG did not differ between groups (*P* = 0.932) nor did G:F differ (*P* = 0.712) on a deads-out basis. However, on a deads-in basis, ADG was numerically greater (*P* = 0.102) in the TCD group vs. TFFE group, and G:F was numerically improved (*P* = 0.114) in the TCD group versus TFFE group. No statistical differences were observed from d 91 to closeout between treatment groups for ADG or G:F (*P* = 0.262 and *P* = 0.114, respectively).

When performance was consolidated from d 0 to closeout and expressed on a deads-in basis, ADG was greater (*P* < 0.033) and G:F improved (*P* < 0.014) in TCD heifers relative to TFFE heifers, as would be expected given the differences in mortality between treatment protocols. Similarly, Sturgess and Renter (2017) observed greater ADG and improved feed efficiency in high-risk beef steers that received tulathromycin metaphylaxis compared to tildipirosin, on a deads-in basis. Higher levels of morbidity and mortality are associated with lower ADG, increased veterinary costs, and less efficient feed conversion (Cernicchiaro et al., 2013; Tennant et al., 2014). These results suggest that mortality differences between treatments are the primary catalyst for improved growth performance.

Average shipping weight did not differ (*P* = 0.842) between experimental groups, which is expressed on a deads-out basis. Finally, closeout economics were calculated for TCD and TFFE treatments and are illustrated in Table 6. Using costs and returns encountered during the course of the study (see footnotes on Table 6), TCD heifers produced $50.78 more income per animal than TFFE heifers. Calf purchase price and value at closeout in this study were $133/45.4 kg BW and $100/45.4 kg BW, respectively. The cost of mortality increases with fed cattle prices, and the importance of using an efficacious metaphylactic antimicrobial becomes greater if incoming cattle are high-risk. It is well-established that treating all high health-risk cattle with metaphylaxis broadly increases expected net return and mitigates return variability (Dennis et al., 2020).

**Table 6.** Closeout economic comparison

Item	TCD^1^	TFFE^2^
Costs^3^		
Animals^4^	334,298	333,793
Metaphylaxis	12,912	11,244
BRD^5^ treatment product	3,321	5,613
BRD treatment labor^6^	980	1,245
Total BRD treatment costs	4,301	6,858
Feed	220,603	210,537
Total	572,114	562,431
Revenue^3^		
Animal value at harvest^7^	574,900	539,880
Net return^8^	2,786	−22,551
TCD vs. TFFE advantage	25,337	–
TCD vs. TFFE advantage per animal	50.78	–

Metaphylaxis with tulathromycin (2.5 mg/kg BW), followed by first BRD treatment with ceftiofur crystalline acid (6.6 mg/kg BW), followed by second BRD treatment with danofloxacin (8 mg/kg BW).

Metaphylaxis with tildipirosin (4 mg/kg BW), followed by first BRD treatment with florfenicol-flunixin meglumine (40 mg/kg BW; 2.2 mg/kg BW), followed by second BRD treatment with enrofloxacin (12.5 mg/kg BW).

U.S. dollars.

Calf purchase price $133/cwt.

Bovine respiratory disease.

$5.00 labor charge for each BRD treatment.

$100/cwt.

Animal value at harvest less total cost.

## CONCLUSION

The results from this study suggest that morbidity, mortality, growth performance, and economics are affected by the BRD antibiotic management protocols used in cattle at high risk for BRD. Tulathromycin metaphylaxis, followed by ceftiofur crystalline acid for first BRD treatment, followed by danofloxacin for second BRD treatment was superior to a protocol consisting of tildipirosin metaphylaxis, followed by florfenicol-flunixin meglumine for first BRD treatment, followed by enrofloxacin for second BRD treatment. A higher mortality rate in cattle receiving the TFFE protocol appeared to be the primary catalyst for diminished net returns when compared to the TCD protocol.
